# Cryo-EM structures reveal upstream DNA interactions within the mitochondrial transcription initiation complex

**DOI:** 10.1016/j.jbc.2026.113611

**Published:** 2026-09-29

**Authors:** Rory E. Sharkey, Caitlin Schroeder, Xiangyu Deng, Jamie Smith, Alfredo J. Hernandez, Yang Gao

**Affiliations:** 1 Department of BioSciences, Rice University, Houston, Texas, USA; 2 Department of Biology, Tufts University, Medford, Massachusetts, USA

**Keywords:** mitochondria, mitochondrial DNA (mtDNA), PolRMT, transcription, TFAM, transcription initiation

## Abstract

Mitochondrial DNA transcription is essential for cellular energy production and is carried out by a streamlined transcription system in which transcription factor A (TFAM), transcription factor B2, and the mitochondrial RNA polymerase (PolRMT) assemble at defined promoters to initiate transcription. Previous structural studies elucidated the core initiation mechanism but relied on truncated promoter templates that excluded upstream regulatory DNA interactions. Here, we present two conformations of mitochondrial transcription initiation complexes assembled on the heavy-strand promote: a TFAM-bound complex with extended upstream DNA and a TFAM-free complex containing short linear DNA. The TFAM-bound structure reveals a transcription-stimulatory interface between PolRMT and the upstream DNA, termed upstream backbone interface, enabled by TFAM-induced promoter bending. Consistent with this structural observation, upstream backbone interface truncation reduces transcription from all mitochondrial DNA promoters, an effect abolished by mutation of the PolRMT interface. In contrast, the TFAM-free structure reveals a transcription-inhibitory interaction of linear upstream DNA with the PolRMT tether helix, which would sterically clash with TFAM binding. Deletion of the tether helix increases off-target transcription, supporting an autoinhibitory role that enhances promoter specificity. Together, these findings reveal how interactions of TFAM and PolRMT with upstream DNA influence activity and specificity of mitochondrial transcription initiation.

Mitochondria orchestrate cellular life and death signals through energy production, immune signaling, and apoptotic pathways 1, 2. Central to these diverse functions is the expression of the mitochondrial genome mitochondrial DNA (mtDNA), a compact and evolutionarily distinct genome housed inside the organelle 3, 4. Transcription of mtDNA results in expression of 13 core proteins involved in oxidative phosphorylation, the key energy-producing pathway, as well as 22 tRNAs, 2 rRNAs, and mtDNA replication primers 3, 4, 5. As such, dysregulation of mitochondrial transcription has been linked to various disorders, including neurodegenerative and cardiovascular diseases, as well as cancer 6, 7, 8, 9.

In stark contrast to the multi-subunit, highly complex nuclear transcription machinery (10), mitochondrial transcription initiation relies on just three proteins 4, 11, 12. Transcription is carried out by PolRMT, a nuclear-encoded single-subunit RNA polymerase, structurally related to the T7 bacteriophage RNAP (13). Unlike the T7 RNAP, transcription initiation by PolRMT requires two transcription factors: transcription factor A (TFAM), which binds to and bends promoter DNA 14, 15, 16, and transcription factor B2 (TFB2M), which interacts with PolRMT near the transcription start site (TSS) to stabilize the transcription bubble 15, 17, 18, 19. Mitochondrial transcription is initiated at three promoter sequences, known as the heavy-strand promoter (HSP), the light-strand promoter (LSP), and the light-strand promoter 2 (LSP2) (20). The HSP is the principal promoter for mitochondrial gene expression as it encodes for 12 of the 13 mRNAs, 14 of the 22 tRNAs, and both of the rRNAs, while LSP and LSP2 encode the remaining RNAs 3, 4, 11. Additionally, regulation of transcription initiation is affected by TFAM-mediated compaction of mtDNA into nucleoids, where tight nucleoid compaction inhibits transcription (21). Furthermore, the noncoding 7S RNA was recently shown to stimulate formation of a transcriptionally-inactive PolRMT dimer (22). While there are far fewer components of mitochondrial transcription relative to nuclear transcription, the mechanisms and interactions influencing mitochondrial transcription initiation are nuanced and require further elucidation.

Despite extensive biochemical and structural analyses of mitochondrial transcription initiation complexes (mtTICs) 15, 23, 24, 25, 26, 27, critical aspects of promoter engagement remain unresolved. Notably, previously solved structures 15, 23, 24 relied on promoter templates derived from the LSP and limited to the −40 to −50 positions relative to the TSS, potentially excluding upstream regulatory DNA interactions. However, DNase-footprinting and cross-linking have shown that mtTICs interact with DNA further upstream (−50 to −60) 20, 28, 29. Consistent with this observation, *in vitro* transcription using longer promoter DNA templates has demonstrated enhanced transcription activity from both LSP and HSP, with the effect being most pronounced for HSP 27, 28. Moreover, the enhanced transcription for HSP was proposed to involve binding of an additional TFAM molecule and looping of the extended DNA (28). Together, these findings suggest that intrinsic elements of the upstream DNA, particularly for the HSP, and PolRMT contribute to transcription enhancement in ways not captured by current structures.

Current structures have established an interaction between TFAM and the PolRMT tether helix 15, 23, 24, 27, suggesting an essential role of TFAM in promoting PolRMT binding and mtTIC assembly. Paradoxically, although TFAM is considered essential for canonical mtTIC formation, biochemical and structural studies have demonstrated that a stable and active transcription complex can form in its absence 23, 25, 28, 30. Further, studies on truncation of the PolRMT N-terminal extension (NTE), which contains the tether helix, have shown enhanced transcription activity in *Drosophila* (31) and increased off-target transcription initiation of mouse PolRMT *in vitro*, suggesting an additional role of the NTE in modulating activity (25). Elucidating the interactions between TFAM, PolRMT, and promoter DNA is required to clarify how these elements influence activity and specificity of transcription initiation.

Here, we present cryo-EM analysis of an mtTIC with an extended HSP template, which revealed two distinct conformations that identify key interactions with the upstream DNA. The first structure, HSP with TFAM, resolves an interaction of PolRMT with extended upstream DNA, which we have termed the upstream backbone interface (UBI). Consistent with the structural interface, templates containing the UBI had enhanced transcript production from all three promoters *in vitro*. Further, mutation of three key lysine residues (K425 E/K428 E/K432 E) at the PolRMT interface ablated UBI-mediated transcription enhancement. The second conformation showed a complex with short, linear upstream DNA engaged by the PolRMT tether helix but without TFAM. Truncation of the tether helix increased transcription initiation on a template lacking the TFAM binding site, suggesting an autoinhibitory mechanism that enforces promoter specificity. Together, our study defines interactions between PolRMT and upstream DNA, which are dependent on TFAM-induced promoter bending, and reveals how these interactions modulate activity and specificity of transcription initiation.

## Results

### Cryo-EM structure of the mitochondrial transcription initiation complex on an extended heavy-strand promoter

Although extended promoter regions have been shown to stimulate mitochondrial transcription initiation (28), the underlying mechanism remains unclear. Previous structural studies employed DNA substrates containing minimal promoter sequences 40 to 50-bp upstream of the TSS 15, 23, 24. To investigate the role of extended promoter DNA in mitochondrial transcription initiation, we performed single-particle cryo-EM analysis of the mtTIC with an extended HSP template. The DNA substrate consisted of the HSP sequence from −60 to +11, with a 7-bp bubble (−4 to +3) generated by mutating the nontemplate strand (Table S1), similar to the mtTIC crystal structure (15) (Fig. 1*A*). The HSP with TFAM mtTIC structure was determined at 3.35 Å resolution (Fig. S1 and Table 1). We were able to resolve TFAM, TFB2M, and PolRMT along with DNA from the −51 to +8 position (Fig. 1*B*), including an extended linear DNA that approaches the N-terminal domain (NTD) of PolRMT (Fig. 1*C*).

**Figure 1:**
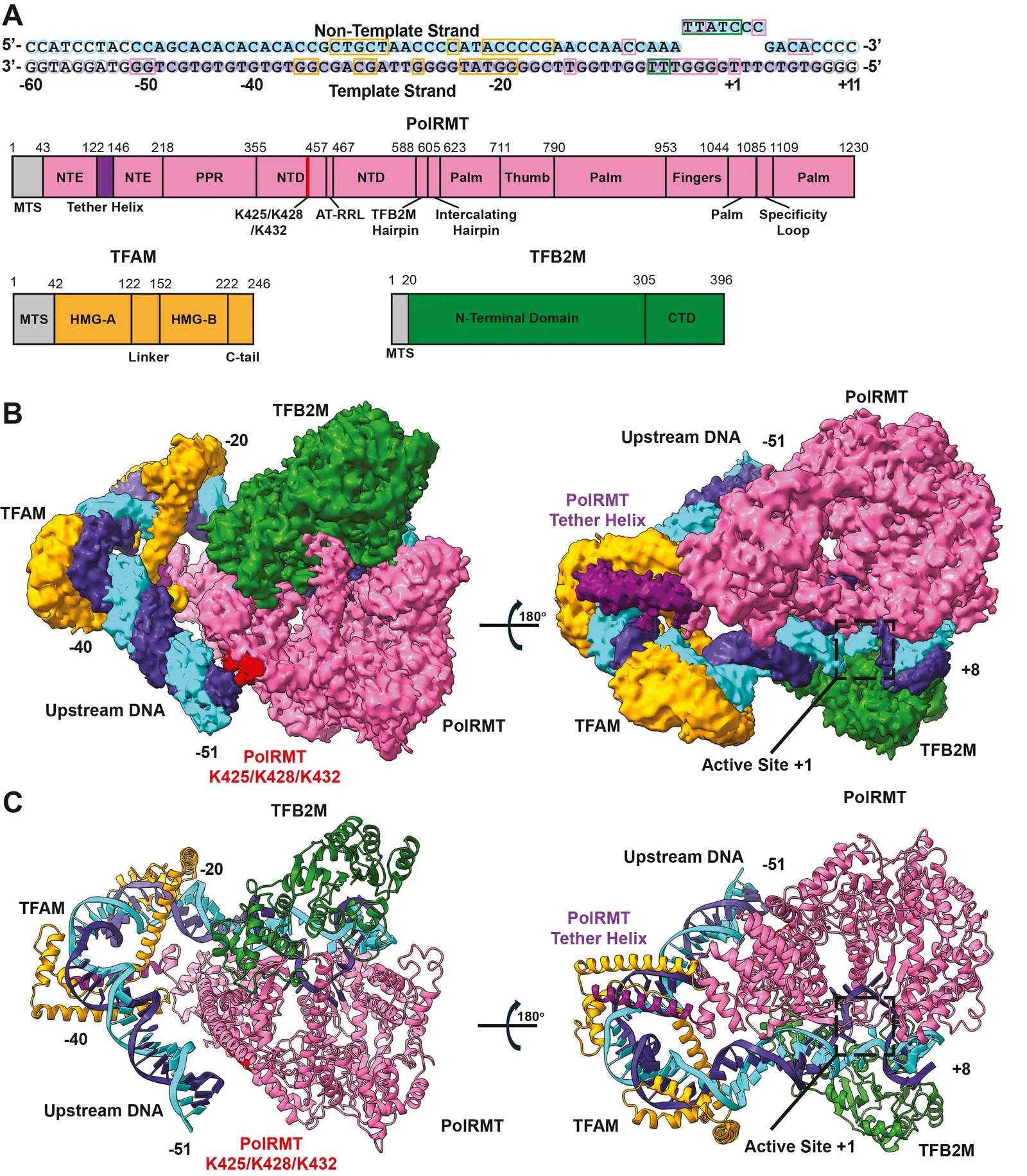
**Cryo-EM structure of the Mitochondrial Transcription Initiation Complex on HSP with extended upstream DNA.***A*, schematic of the HSP bubble DNA substrate, TFAM, TFB2M, and PolRMT. The DNA substrate nontemplate strand and template strand are shown in *cyan* and slate, respectively, and *shaded circle*s represent the resolved bases, bases that are not visible are represented by *hollow circles*. Protein-DNA contacts (<4 Å) are indicated by *pink, green* and *orange boxes* for PolRMT, TFB2M, and TFAM, respectively. PolRMT is shown in *pink* and divided into its domains: MTS (not included in expression constructs), N-terminal extension, tether helix in *purple*, pentatricopeptide repeat domain, N-terminal domain, residues K425, K428, and K432 in *red*, AT-rich recognition loop, TFB2M-interacting hairpin (TFB2M hairpin), palm, thumb, and specificity loop. TFAM is shown in orange with the MTS, high mobility group box *A*, linker, high mobility group-B, and C-terminal tail. TFB2M is shown in green with the MTS, N-terminal domain, and C-terminal domain. *B*, *left*: cryo-EM density map of the mtTIC on the HSP substrate showing the extended upstream DNA with proteins and DNA colored as in *(A)*. *Right*: 180° rotation around the X-axis showing the PolRMT active site and downstream DNA. *C*, cartoon representation of DNA and proteins shown in *(B)*. MTS, mitochondrial targeting sequence; PolRMT, mitochondrial RNA polymerase; HSP, heavy-strand promoter; TFAM, transcription factor A; TFB2M, transcription factor B2; MTS, mitochondrial targeting sequence.

**Table 1:** Cryo-EM data collection, model refinement, and validation

**Name**	HSP TIC with TFAM	HSP TIC without TFAM
Data collection and processing		
EMDB ID	78,147	78,146
PDB ID	37FK	37FJ
Microscope	Titan Krios G4i (Δ)	Titan Krios G4i (Δ)
Detector	Falcon 4i	Falcon 4i
Magnification	146,000	146,000
Voltage (kV)	300	300
Electron exposure (e^-^/Å^2^)	50	50
Defocus range (μm)	−0.8 to −2.0	−0.8 to −2.0
Pixel size (Å)	0.958	0.958
Number of movies	27,688	27,688
Initial particles (no.)	11,635,618	32,940,939
Final particles (no.)	200,000	446,000
Map resolution (Å) FSC threshold = 0.5	3.70	3.40
Map resolution (Å) FSC threshold = 0.143	3.35	2.87
Refinement		
No. of atoms	15,210	12,272
No. of residues	-	-
Protein	1585	1352
Nucleic acid	118	70
RMSDs	-	-
Bond lengths (Å)	0.004	0.004
Bond angles (°)	0.889	0.848
Validation		
MolProbity score	1.19	1.53
Clashscore	2.30	6.82
Rotamer outlier (%)	0.00	0.08
Ramachandran plot (%)	-	-
Favored	96.95	97.17
Allowed	3.05	2.83
Outliers	0.00	0.00
B-Factors		
Iso/Ansio (#)	15,210/0	12,272/0
Min/max/mean	-	-
Protein	26.07/347.10/128.48	79.007/387.37/147.04
Nucleotide	93.43/453.69/272.43	136.28/624.63/319.06
Model *versus* Data		
CC (mask)	0.79	0.71
CC (box)	0.85	0.84
CC (peaks)	0.74	0.67
CC (volume)	0.78	0.71
Q-score	0.36	0.351

The PolRMT and TFB2M core in our transcription initiation complex adopts a similar conformation to that observed in previous structures 15, 23, 24. Alignment of PolRMT and TFB2M Cα atoms results in an RMSD of 1.1 Å over 1100 residues to the HSP crystal structure (PDB: 6ERQ) and 0.8 Å over 889 residues to an LSP structure solved by cryo-EM (PDB: 9MN5) (Table S2) 15, 23, 24. The PolRMT fingers domain adopts a clenched conformation in our structure, which is consistent with other structures that do not have an incoming NTP bound 15, 24 (Fig. S2*A*). However, TFAM is rotated by 15° in our structure compared to the HSP crystal structure (PDB: 6ERQ) and 11° compared to the LSP cryo-EM structure (PDB: 9MN5), suggesting flexibility of TFAM within the mtTIC 15, 23, 24 (Fig. S2*B* and Table S2).

### PolRMT interacts with the upstream DNA backbone during transcription initiation

In our mtTIC structure, clear density can be observed for the HSP UBI, which forms an additional interaction interface with PolRMT. Specifically, K425, K428, and K432, which are located in a helix of the PolRMT NTD, contact the DNA phosphate backbone through electrostatic interactions (Fig. 2*A*). While our structure reveals this interaction on the HSP, DNase footprinting experiments have suggested that the interaction occurs on all three mitochondrial promoters (20). To test the role of the UBI in transcription initiation, we designed long DNA templates including the UBI to the −70 position and truncated templates that ended at the −40 position from all three promoters (Fig. 2*B*). An *in vitro* transcription assay using the reconstituted mtTIC with PolRMT titration was employed to measure transcript production from each DNA substrate. On the LSP, the truncated template had ∼50% lower transcript production (Fig. 2*C*). Similarly, template truncation led to a decrease in transcript production for both HSP and LSP2, with a 20% and 50% reduction, respectively (Fig. 2*D* and E). Together, these results show that the PolRMT NTD interacts with the UBI and that this interaction is important for transcription initiation at all three mitochondrial promoters.

**Figure 2:**
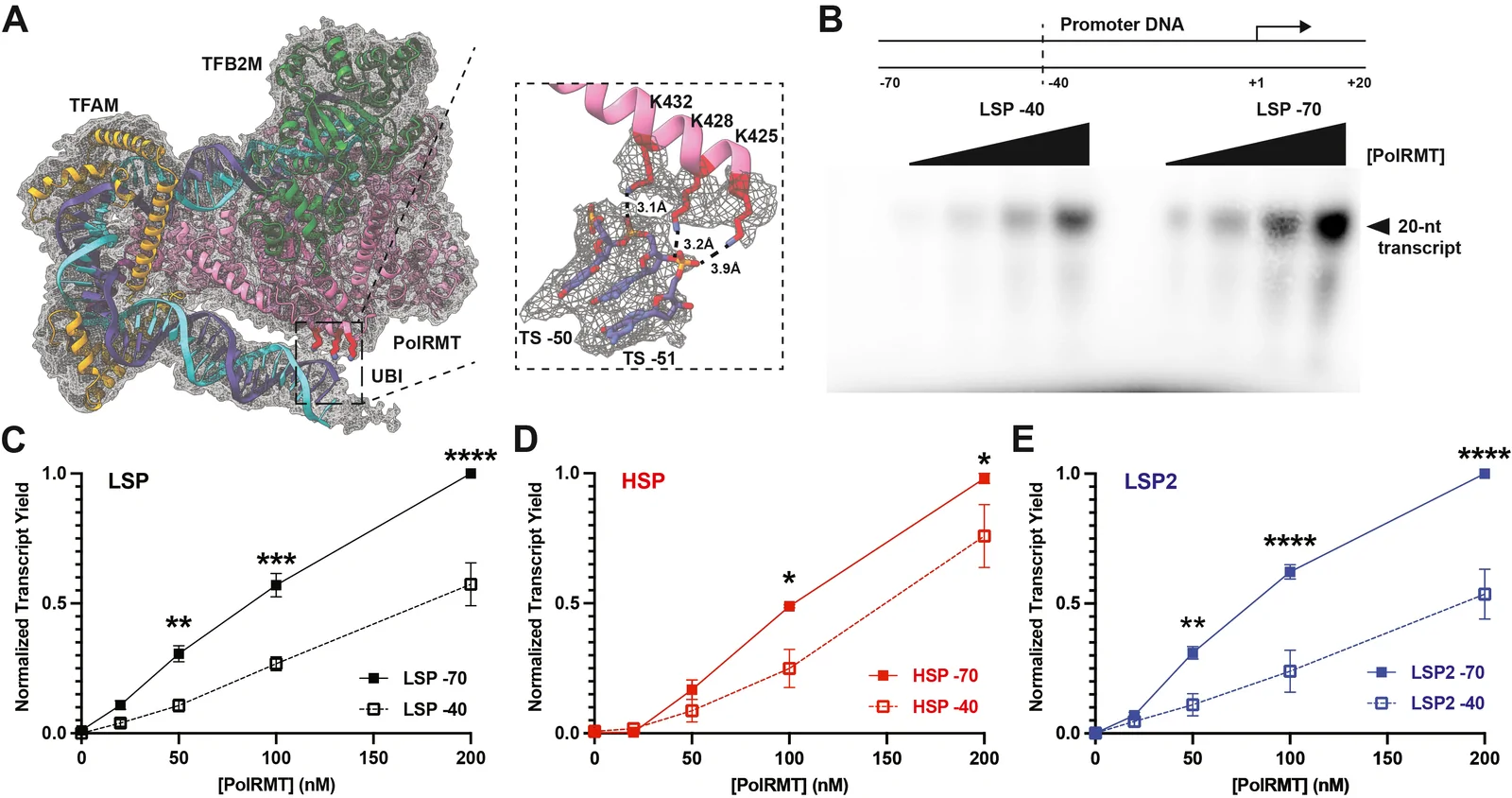
**PolRMT interacts with the UBI during transcription initiation.***A*, cryo-EM density map highlighting the interaction of PolRMT K425, K428, and K432 with the UBI, specifically, the DNA backbone of the template strand at bases −50 and −51. *B*, DNA substrate design for transcription initiation assays with the short template extending to the −40-bp and long template to the −70-bp. Representative gel image of PolRMT titration from 0 to 200 nM with the −40 and −70-bp LSP DNA template. *C*, quantification of transcript production from the LSP with −40 and −70-bp template. *D*, quantification of transcript production from the HSP with −40 and −70-bp template. *E*, quantification of transcript production from the LSP2 with −40 and −70-bp template. Mean and SEM were determined from 3 independent replicates. Significance was determined using a 2-way ANOVA test with Šídák’s multiple comparisons test. Significance: ∗*p* < 0.05, ∗∗*p* < 0.01, ∗∗∗*p* < 0.001, ∗∗∗∗*p* < 0.0001. Statistical analysis: *C*, 2-way ANOVA: [PolRMT] factor F(4,8) = 206.5, *p* < 0.0001, DNA substrate Factor F(1,2) = 69.53, *p* = 0.0141, [PolRMT] x DNA substrate F(4,8) = 17.97, *p* = 0.0005; Šídák’s multiple comparisons test adjusted *p* values: 50 nM PolRMT *p* = 0.0052, 100 nM PolRMT *p* = 0.0003, 250 nM PolRMT *p* < 0.0001. D) 2-way ANOVA: [PolRMT] factor F(4,8) = 243.2, *p* < 0.0001, DNA substrate Factor F(1,2) = 5.055, *p* = 0.1538, [PolRMT] x DNA substrate F(4,8) = 3.580, *p* = 0.0588; Šídák’s multiple comparisons test adjusted *p* values: 100 nM PolRMT *p* = 0.0285, 250 nM PolRMT *p* = 0.0416. *E*, 2-way ANOVA: [PolRMT] factor F(4,8) = 141.6, *p* < 0.0001, DNA substrate Factor F(1,2) = 32.67, *p* = 0.0293, [PolRMT] x DNA substrate F(4,8) = 27.61, *p* < 0.0001; Šídák’s multiple comparisons test adjusted *p* values: 50 nM PolRMT *p* = 0.0049, 100 nM PolRMT *p* < 0.0001, 250 nM PolRMT *p* < 0.0001. UBI, upstream backbone interface; PolRMT, mitochondrial RNA polymerase; LSP, light-strand promoter; HSP, heavy-strand promoter.

### Mutation of PolRMT residues K425, K428, and K432 abolishes UPR enhancement of transcript yield

To directly test the role of PolRMT residues K425, K428, and K432 in mediating interaction with the UBI, we mutated all three lysines to glutamate (K425 E/K428 E/K432 E; 3 KE) to disrupt electrostatic interactions with the negatively charged DNA backbone. We then evaluated transcription initiation by the 3 KE mutant using both long and truncated DNA templates. In contrast to WT PolRMT, transcript yield with the 3 KE mutant was largely insensitive to promoter truncation (Fig. 3, *A*–*C*). For LSP, the 3 KE mutant produced slightly greater transcript yield from the shorter DNA template, opposite to WT PolRMT (Fig. 3*A*). On both HSP and LSP2, 3 KE PolRMT produced similar transcript yield from both templates across all PolRMT concentrations tested (Fig. 3*B* and C). These results confirmed that the interaction between PolRMT and the UBI is mediated primarily by the interaction of PolRMT K425, K428, and K432 with the DNA backbone.

**Figure 3:**
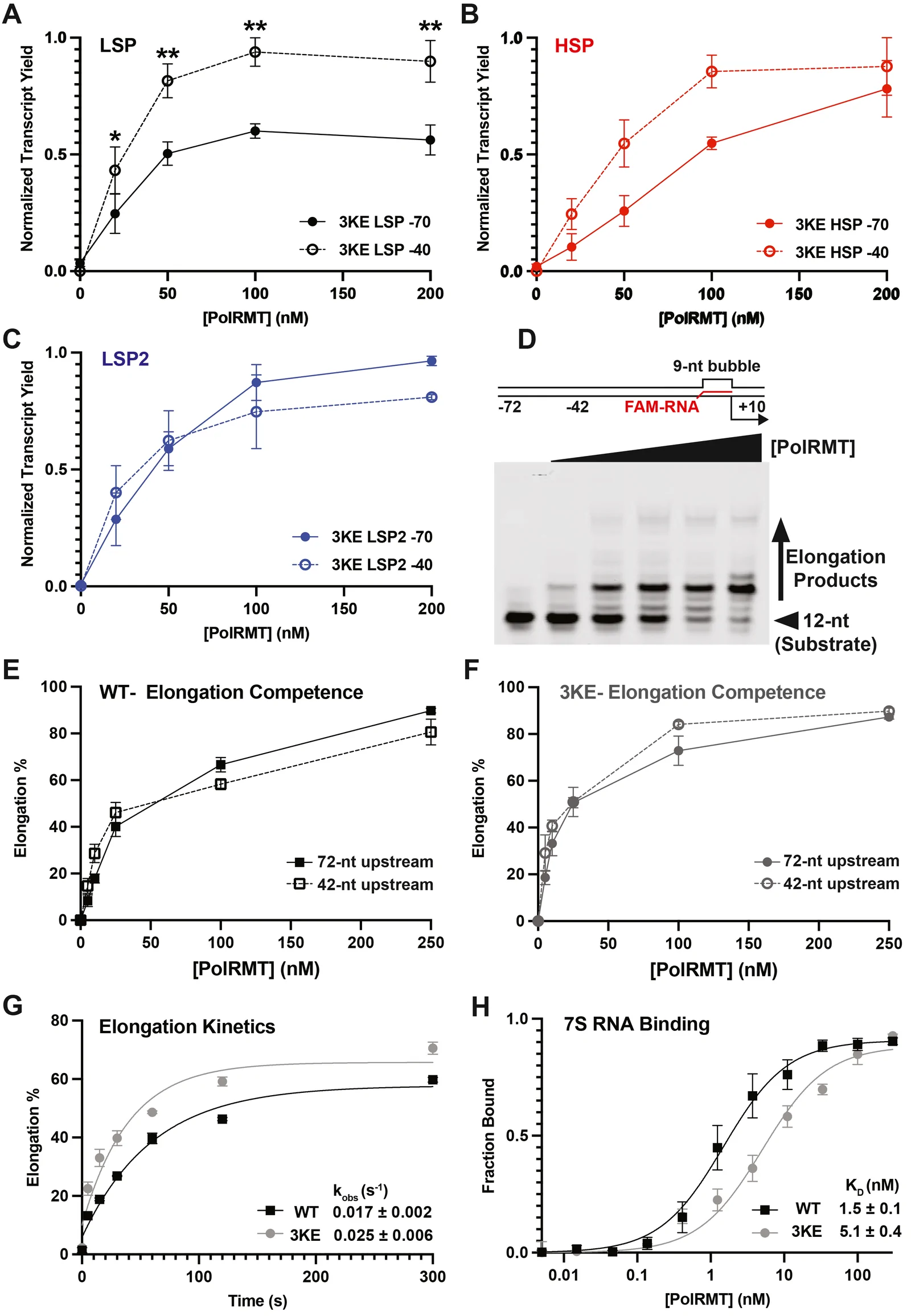
**Mutation of PolRMT K425/K428/K432 abolishes the enhancement of transcript yield by the UBI.***A*, quantification of PolRMT K425 E/K428 E/K432 E (3 KE) transcript production from the LSP with −40- and −70-bp template. *B*, quantification of PolRMT 3 KE transcript production from the HSP with −40- and −70-bp template. *C*, quantification of PolRMT 3 KE transcript production from the LSP2 with −40- and −70-bp template. Mean and SEM were determined from 3 independent replicates. Significance was determined using a 2-way ANOVA test with Šídák’s multiple comparisons test. Significance: ∗∗*p* < 0.01. *D*, elongation substrate used for promoter-free transcription elongation assays. The 12-nt FAM-labeled RNA is shown in red and the length of upstream DNA is indicated as either 42-nt or 72-nt. Representative gel of promoter-free transcription elongation assay with titration of PolRMT from 0 to 250 nM. The unelongated 12-nt substrate and elongation products greater than 12-nt are indicated. *E*, quantification of promoter-free transcription elongation competence assay by WT PolRMT on the −72- and −42-nt elongation scaffolds. *F*, quantification of promoter-free transcription elongation competence assay by 3 KE PolRMT on the −72- and −42-nt elongation scaffolds. Mean and SEM were determined from 3 independent replicates. *G*, quantification of promoter-free transcription elongation kinetics assay from −42-nt elongation scaffold with nonspecific DNA trap. Mean and SEM were determined from 2 independent replicates. *H*, quantification of filter binding assays for PolRMT WT and 3 KE binding to 7S RNA. Mean and SD were determined from 5 independent replicates. Statistical analysis: *A)* 2-way ANOVA: [PolRMT] factor F(4,8) = 33.21, *p* < 0.0001, DNA substrate Factor F(1,2) = 33.87, *p* = 0.0283, [PolRMT] x DNA substrate F(4,8) = 9.048, *p* = 0.0046; Šídák’s multiple comparisons test adjusted *p* values: 20 nM PolRMT *p* = 0.0396, 50 nM PolRMT *p* = 0.0018, 100 nM PolRMT *p* = 0.0011, 250 nM PolRMT *p* = 0.0011. B) 2-way ANOVA: [PolRMT] factor F(4,8) = 236.3, *p* < 0.0001, DNA substrate Factor F(1,2) = 8.596, *p* = 0.0993, [PolRMT] x DNA substrate F(4,8) = 1.046, *p* = 0.4414. C) 2-way ANOVA: [PolRMT] factor F(4,8) = 32.18, *p* < 0.0001, DNA substrate Factor F(1,2) = 0.6286, *p* = 0.5110, [PolRMT] x DNA substrate F(4,7) = 3.377, *p* = 0.0768. UBI, upstream backbone interface; PolRMT, mitochondrial RNA polymerase; LSP, light-strand promoter; HSP, heavy-strand promoter; LSP2, light-strand promoter 2.

To test whether this interaction may also affect transcription elongation competence or kinetics, we employed a promoter-independent transcription elongation assay. The elongation scaffold was designed with a 9-nt bubble between the NT and TS where the labeled RNA was paired with the TS, as in previous studies 32, 33, and an upstream DNA length of either 40 or 72-bp (Fig. 3*D*). The length of upstream DNA had no significant impact on transcription elongation competence for either WT or the 3 KE mutants (Fig. 3*E* and F). Additionally, the mutant maintained similar elongation kinetics to the WT PolRMT (Fig. 3*G*). Taken together, this suggests that the interaction of PolRMT with extended upstream DNA occurs only during transcription initiation, likely facilitated by TFAM bending of promoter DNA into a U-shape.

Previous studies suggested that PolRMT binding to 7S RNA negatively regulates transcription initiation (22). The structure of a transcriptionally-inactive PolRMT dimer bound to 7S RNA suggested that, along with several other positively charged residues, K425, K428, and K432 may contact 7S RNA (22). To probe whether the positively charged residues K425, K428, and K432 may contribute to transcription regulation *via* RNA interactions, we evaluated binding of PolRMT to 7S RNA using a filter binding assay. We saw a 3-fold decrease in binding affinity in the 3 KE mutant compared to WT (Fig. 3*H*). Further, we observed that WT PolRMT has a strong preference for 7S RNA compared to a control RNA (unrelated sequence of similar length) in both direct binding and binding competition assays (Fig. S3). These findings suggest that K425, K428, and K432 contribute not only to transcription initiation but may also contribute to regulation *via* the extensive PolRMT–7S RNA binding interface.

### Cryo-EM structure of the mitochondrial transcription initiation complex without TFAM

During cryo-EM data processing (Fig. S1 and Table 1), we observed a second class of mtTIC in which TFAM was not present and short linear upstream DNA was observed (Figs. 4*A* and S4). This structure was resolved to 2.87 Å resolution, and it lacked density for DNA upstream of position −24 and the PolRMT NTE, residues 44–121 and 143–217 (Fig. 4*A*). Notably, we observed electron density adjacent to the short, linear upstream DNA that can accommodate the PolRMT tether helix, which contains several positively charged residues and could interact with the DNA backbone (Fig. S5). Aside from the tether helix orientation and linear upstream DNA, the PolRMT and TFB2M core of this structure was similar to our structure containing TFAM with an RMSD of 1.0 Å over 1293 residues.

**Figure 4:**
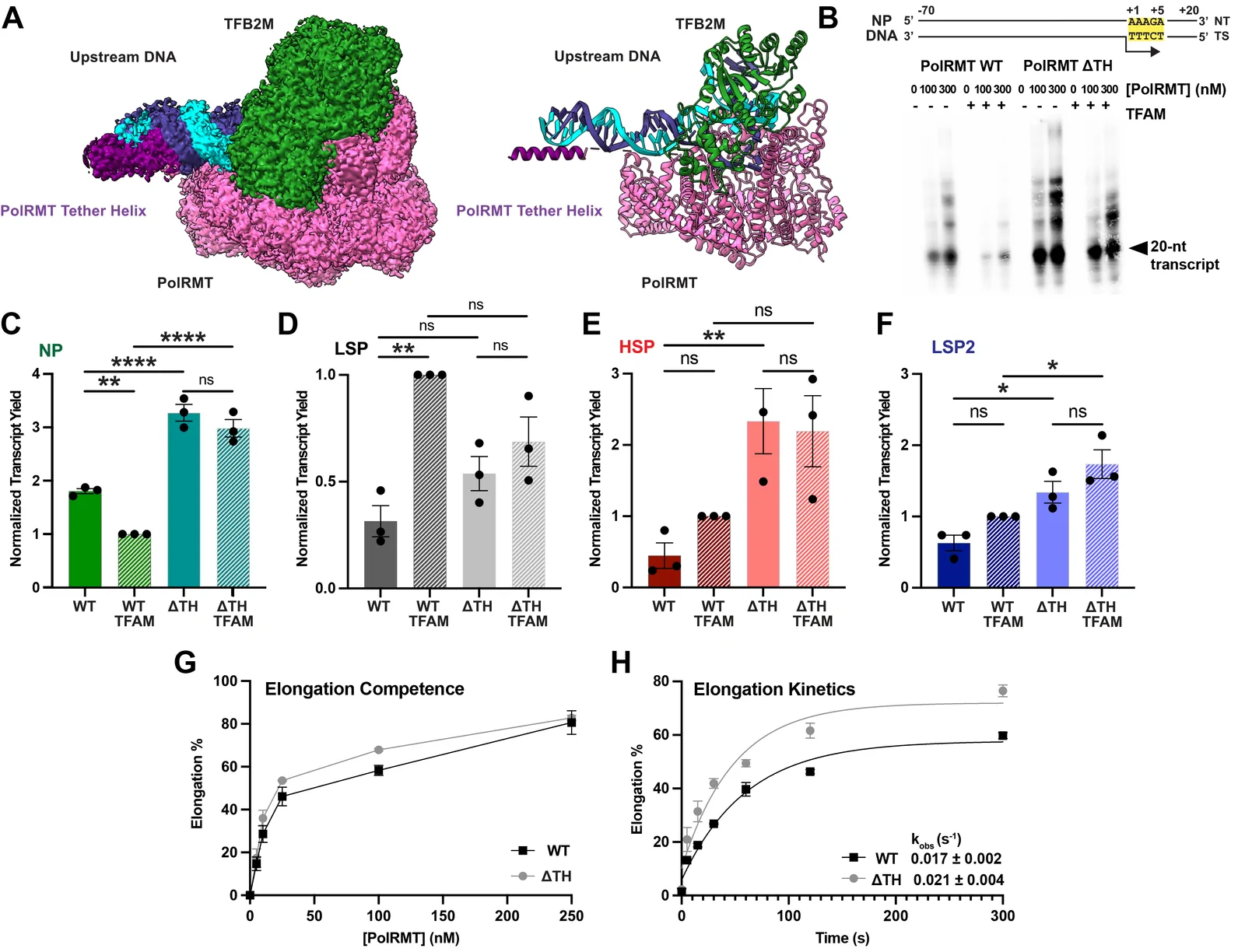
**The PolRMT tether helix has an autoinhibitory effect on nonspecific transcription initiation and initiation from the HSP and LSP2.***A*, *left*: cryo-EM density map of the mtTIC without TFAM, and a short linear upstream DNA with adjacent electron density that accommodates the PolRMT tether helix. Right: Cartoon representation of DNA and proteins colored as in (Fig. 1*A*). *B*, DNA sequence for NP transcription initiation template that contains the +1 to +5 sequence conserved amongst all three promoters *(highlighted yellow)* and is derived from the mtDNA genome, positions 4211 to 4300 (3). Representative gel image of transcription initiation by PolRMT WT or ΔTH in the presence or absence of TFAM on the NP DNA template with the 20-nt transcription product indicated. Transcription reactions contained 20 nM DNA template, 100 nM TFB2M, 0 or 60 nM TFAM, and 0, 100, or 300 nM PolRMT. *C*, quantification of transcript production from the NP DNA substrate. *D*, quantification of transcript production from the LSP. *E*, quantification of transcript intensity from the HSP. *F*, quantification of transcript production from the LSP2. *G*, quantification of promoter-free transcription elongation by WT and ΔTH PolRMT on a −42-nt elongation scaffold. Mean and SEM were determined from 3 independent replicates. *H*, quantification of promoter-free transcription elongation kinetics assay from −42-nt elongation scaffold with nonspecific DNA trap. Mean and SEM were determined from 2 independent replicates. Significance was determined using a 1-way ANOVA test with Tukey’s multiple comparisons test. Significance: ∗*p* < 0.05, ∗∗*p* < 0.01, ∗∗∗∗*p* < 0.0001. Statistical analysis: *C*, ANOVA: F(3,8) = 83.39, *p* < 0.0001; Tukey's multiple comparisons test adjusted *p* values: WT *versus* WT TFAM = 0.0051, WT *versus* ΔTH < 0.0001, WT TFAM *versus* ΔTH TFAM < 0.0001. *D*, ANOVA: F(3,8) = 13.17, *p* = 0.0018; Tukey's multiple comparisons test adjusted *p* values: WT *versus* WT TFAM = 0.0013. *E*, ANOVA: F(3,8) = 6.893, *p* = 0.0131; Tukey's multiple comparisons test adjusted *p* values: WT vs ΔTH = 0.0215. *F*, ANOVA: F(3,8) = 11.77, P + 0.0026; Tukey's multiple comparisons test adjusted *p* values:, WT versus ΔTH = 0.0265, WT TFAM *versus* ΔTH TFAM = 0.0227. PolRMT, mitochondrial RNA polymerase; LSP, light-strand promoter; HSP, heavy-strand promoter; LSP2, light-strand promoter 2; NP, non-promoter.

### The tether helix of PolRMT influences promoter selection through an autoinhibitory effect

The apparent density for the PolRMT tether helix near the linear upstream DNA suggested a possible role in transcription initiation in the absence of TFAM. To test this, we generated a PolRMT mutant lacking the tether helix (residues 122–146) (ΔTH). Previous work on mouse PolRMT demonstrated that truncation of the entire NTE (including the tether helix) led to off-target transcription initiation (25). To test nonpromoter transcription (NP) initiation, we employed a DNA fragment from the mtDNA genome that contains the conserved AAAGA sequence, which is present at positions +1 to +5 on all three promoters and occurs at over 30 locations throughout the mtDNA genome but lacks the promoter TFAM binding site (Fig. 4*B*). We performed *in vitro* transcription assays to test the transcript yield from WT and ΔTH PolRMT in both the presence and absence of TFAM. The ΔTH mutant had significantly higher NP transcript yield than WT, independent of the presence of TFAM (Fig. 4*C*). Inclusion of TFAM significantly decreased NP transcription of WT PolRMT, while only a slight, but not significant, reduction was observed for ΔTH (Fig. 4*C*). Further, we used this assay to test each of the three mitochondrial promoters. On LSP, inclusion of TFAM increased transcript yield by WT PolRMT, while ΔTH had similar yield to WT in the absence of TFAM and was not significantly enhanced by the presence of TFAM (Fig. 4*D*). On HSP, ΔTH had significantly greater TFAM-independent transcription yield than WT, while TFAM-dependent transcription yield was slightly higher than WT (Fig. 4*E*). On LSP2, ΔTH had greater transcript production in both the presence and absence of TFAM compared to WT (Fig. 4*F*). A similar trend was also observed when the PolRMT concentration was increased (Fig. S6). Additionally, promoter-free transcription elongation competence and kinetics were nearly identical for WT and ΔTH PolRMT (Fig. 4*G* and H). Taken together, these results suggest that the PolRMT tether helix has an autoinhibitory role in transcription initiation from the HSP and LSP2 promoters, and that it contributes to the specificity of transcription initiation by limiting nonpromoter initiation.

## Discussion

Human mtDNA transcription initiation is a streamlined, three-component system that relies on the coordinated activities of PolRMT, TFB2M, and TFAM. TFAM is unique among transcription factors in that it also functions as the principal mitochondrial genome-packaging protein (34). Here, we identify critical interactions between three positively charged lysine residues of PolRMT and the DNA backbone of the UBI. Our structural and biochemical analyses align well with findings from Morozov *et al.* (27), suggesting that these interactions are enabled by the TFAM-induced U-shaped DNA architecture promoting productive assembly of the mtTIC. Consistent with this, the UBI enhances transcription initiation on all three mitochondrial promoters, and this stimulatory effect is abolished by mutations at the PolRMT interface that contacts the UBI. Importantly, this interaction is specific to initiation, as elongation proceeds independently of TFAM and is unaffected by these mutations. Notably, the same PolRMT interface also contributes to binding of the transcription-inhibitory 7S RNA (22), as mutations reduce 7S RNA binding, suggesting a shared binding surface that may influence transcription regulation.

Because TFAM binds DNA with limited sequence specificity and is highly abundant in mitochondrial nucleoids, a key question is how off-target transcription is avoided. Our findings suggest a multilayered mechanism in which template DNA, TFAM-mediated architecture, and PolRMT–upstream DNA interactions cooperate to promote specific transcription initiation (Fig. 5*A*). *In vitro* transcription assays show that truncation of the DNA template to eliminate the UBI reduces transcription activity. In contrast, deletion of the PolRMT tether helix maintains high activity but compromises promoter specificity, resulting in increased nonpromoter transcription. Both activity and specificity are reduced in the absence of TFAM. TFAM itself contributes to promoter specificity as mitochondrial promoter sequences support greater DNA bending and complex stability than nonpromoter DNA 35, 36, while high TFAM occupancy of mtDNA directly inhibits PolRMT access and transcription (21). Together, these features establish a coincidence-detection mechanism in which promoter sequence, TFAM-mediated DNA bending, and PolRMT conformational control must align to permit specific and efficient initiation.

**Figure 5:**
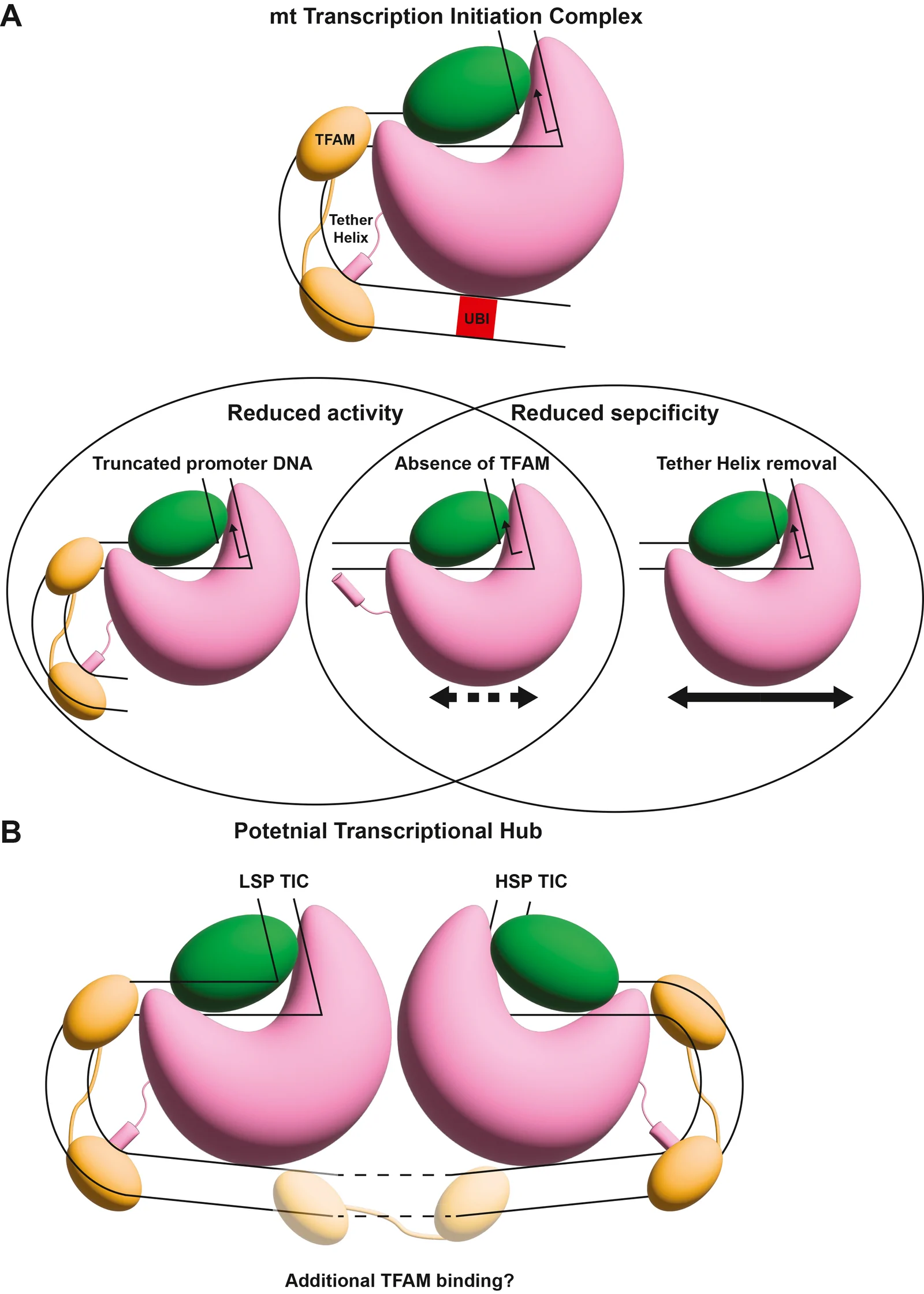
**Model for protein–DNA interactions influencing mitochondrial transcription initiation.***A*, *(Top)* the canonical mtTIC exhibits high activity and specificity with contributions from TFAM bending DNA into a U-turn shape, sequestering the PolRMT tether helix and facilitating the PolRMT–UBI interaction. *(Bottom left)* functional assays showed that truncation of the UBI reduced transcription activity. *(Bottom center)* TFAM-independent transcription shows both reduced activity and specificity. *(Bottom right)* Deletion of the PolRMT tether helix maintains transcription activity but compromises promoter specificity leading to nonpromoter initiation. *B*, the mtTIC assembled at both the LSP and HSP ends of the inter-promoter region, with dashed lines to indicate the potential for additional TFAM binding and topological rearrangement. LSP, light-strand promoter; HSP, heavy-strand promoter; UBI, upstream backbone interface.

The PolRMT N-terminal tether helix provides an additional layer of transcriptional control within this mechanism. In the absence of TFAM, we observe the highly positively charged tether helix positioned near the upstream DNA, which remains in a linear conformation. This arrangement would sterically clash with TFAM-induced U-shaped DNA bending, suggesting that tether helix–DNA engagement is mutually exclusive with productive TFAM binding. Consistent with an autoinhibitory effect, deletion of the tether helix increases nonpromoter transcription as well as both TFAM-dependent and TFAM-independent initiation from the HSP and LSP2. The nonspecific engagement of DNA by the tether helix may inhibit PolRMT activity by impairing its ability to scan for the specific TSS. This is analogous to observations of p53 and other DNA-binding proteins that have both specific and nonspecific DNA interactions 37, 38. Alternatively, tether helix–DNA binding may disfavor conformations required for TSS melting 19, 39, 40. Furthermore, the tether helix may adopt intramolecular contacts within PolRMT that reduce nonspecific DNA binding.

The presence of TFAM at canonical promoter sites prevents the tether helix from interacting with upstream DNA. Instead, the tether helix forms electrostatic interactions with an acidic surface of TFAM (Fig. S5), thereby preventing autoinhibition. This TFAM-dependent repositioning of the tether helix provides a potential mechanism by which TFAM occupancy could regulate PolRMT activity as a function of TFAM concentration (21). Such regulation may be particularly relevant at mitochondrial promoters or nucleoids with reduced TFAM occupancy (41), where TFAM-independent transcription initiation may become more accessible. Although the precise mechanism remains to be defined, our data support a model in which the tether helix suppresses spurious initiation and is repositioned only when proper TFAM-dependent promoter architecture is established. This architectural transition may also have broader implications for how TFAM organizes the mitochondrial promoter region and coordinates transcription from the two divergent promoters.

The HSP and LSP in the mitochondrial genome are separated by a 153-bp noncoding region. Previous footprinting studies suggested that multiple TFAM molecules may bind within this region to stimulate transcription from both promoters (28). However, in available TIC structures 15, 23, 24, TFAM-induced U-shaped DNA bending positions the immediate upstream DNA in close proximity to the PolRMT surface, such that additional TFAM binding at positions −30 to −60 would sterically clash. Instead, the ∼30-bp region between the two UBIs could accommodate additional TFAM occupancy. Additional TFAM binding at these distal sites may further remodel the noncoding region, potentially bringing the LSP and HSP initiation complexes into spatial proximity (Fig. 5*B*). This could generate a transcriptional hub that coordinates promoter usage and enables regulatory crosstalk, similarly to transcription by yeast RNA Pol II, which forms a dimer of the polymerase-mediator complexes simultaneously on two divergent promoters (42). While PolRMT dimerization *via* 7S RNA inhibits transcription (22), a different interface was found for dimerization of the apo protein (23), and it is unknown whether a higher-order organization of both promoters may involve transcriptionally productive PolRMT dimerization. Defining this higher-order topology will require future structural and biochemical studies aimed at visualizing multi-TFAM assemblies and both LSP and HSP TICs on extended promoter templates.

Evolutionarily, the mitochondrial transcription machinery has undergone substantial diversification. PolRMT orthologues are broadly distributed across eukaryotes, whereas direct TFAM and TFB2M orthologues are primarily found in animals (43), suggesting that the three-component transcription initiation system is predominantly an animal feature. Fungi instead use the mitochondrial RNA polymerase Rpo41 together with mitochondrial transcription initiation factor Mtf1 44, 45, 46, while plants generally rely on two mitochondrially targeted RNA polymerases, RPOTm and RPOTmp (47). The fungal protein Abf2p, which contains two HMG domains and primarily functions in mitochondrial genome packaging, represents a functional counterpart to TFAM but does not serve as a mitochondrial transcription initiation factor (48). Thus, genome packaging and transcription are coupled through distinct molecular strategies in different eukaryotic lineages.

Our comparative analysis further revealed substantial evolutionary variation in the N-terminal regions of PolRMT. Pentatricopeptide repeat (PPR) domain-containing mitochondrial RNA polymerases were predominantly found in animals, consistent with the emergence of the animal-type mitochondrial transcription machinery. Among the animal PolRMT sequences analyzed, 68% contained a PPR domain, whereas sequences lacking a PPR domain were found primarily in nematodes and arthropods. Strikingly, 97% of PPR-domain-containing sequences also contained either a patch enriched in basic residues or a putative tether helix (Fig. S7, *A* and *B*). Such clusters of basic residues are frequently involved in protein–protein and protein–nucleic acid interactions 49, 50, 51. This supports the conservation of the electrostatic interaction observed in our structures amongst vertebrates. The widespread occurrence of these basic patches is consistent with the conservation of the electrostatic interaction observed in our structures across vertebrate PolRMTs. Supporting this model, helix 3 of the TFAM high mobility group (HMG)-B domain is enriched in negatively charged residues in animals relative to fungi containing ABF2-like proteins (Fig. S7*C*), complementing the positively charged surface of the PolRMT tether helix observed in our structures. While the lysine residues at positions 425, 428, and 432 are not strictly conserved among all animals, positively charged residues at positions 425 and 432 are present in 61% and 75% of mammalian sequences, respectively, suggesting that the UBI may have evolved as a means of transcriptional regulation in mammals (Table S4). Additionally, we have provided tables in the Supporting Information quantifying the sequence conservation and variance at each residue position for PolRMT, TFAM, and TFB2M (Tables S4–S6), which may be a useful resource for further analysis. Together, these findings suggest that the tight coupling between mitochondrial genome organization and transcription initiation has evolved through lineage-specific adaptations rather than through a single conserved mechanism.

In summary, our study demonstrates that extended upstream DNA directly engages PolRMT to enhance transcription initiation, while the tether helix can exert an autoinhibitory effect that enforces promoter specificity. By integrating structural and biochemical analyses, we provide a refined model in which TFAM-mediated DNA architecture and PolRMT–DNA interactions cooperate to influence transcription initiation. More broadly, these findings illustrate how modular extensions appended to a bacteriophage-like RNA polymerase can introduce regulatory complexity into a streamlined transcription apparatus. Future studies aimed at defining higher-order promoter DNA organization and regulatory RNA interactions will further clarify how mitochondrial transcription is dynamically tuned under physiological and pathological states.

## Experimental procedures

### Cloning and expression of TFAM, TFB2M, and PolRMT variants

Human TFAM (residues 43–246) with an N-terminal 6X-His tag and PreScission protease cleavage sequence was inserted into the pET-28p vector *via* In-fusion cloning (Takara Bio). Expression was performed in *Escherichia. coli* BL21(DE3) and cultures were grown in LB at 37 °C until reaching optical density (OD600) of 0.6 to 0.8. Cultures were cooled on ice for 30 min. Then, expression was induced with 1 mM IPTG, and cultures were incubated in a shaker at 20°C for 18 h.

Human TFB2M (residues 21–396) was inserted into the pET-SUMO vector with an N-terminal 6X-His-SUMO2 tag *via* In-fusion cloning. Expression was performed in *Escherichia. coli* BL21(DE3) and cultures were grown in LB at 37 °C to OD600 = 0.6 to 0.8. Cultures were cooled on ice for 30 min. Then, expression was induced with 0.5 mM IPTG, and cultures were incubated in a shaker at 16 °C for 18 h.

Human PolRMT WT (residues 44–1230) was inserted into the pMAL-c6T (NEB) vector with an N-terminal 6X-His-MBP tag and PreScission protease cleavage sequence *via* In-fusion cloning. The vector was transformed into ArcticExpress (Agilent) cells containing the pTF16 plasmid, (Takara Bio) expressing TriggerFactor to enhance solubility. Cultures were grown in LB containing 0.5 mg/ml L-Arabinose and 20 g/L glucose at 37 °C to OD600 = 0.3. Cultures were cooled on ice for ∼2 h before inducing expression with 0.1 mM IPTG, then shaking continued at 10 °C for ∼40 h.

The K425 E/K428 E/K432 E (3 KE) PolRMT mutant construct was generated from the plasmid containing WT *via* In-fusion cloning. The tether helix truncation mutant (deletion of PolRMT residues 122–146 [ΔTH]) was generated *via* inverse PCR and the KLD enzyme mix (NEB) (primers included in Table S1).

### Purification of TFAM, TFB2M, and PolRMT variants

TFAM was purified by first resuspending cell pellets in buffer N1 (20 mM Tris pH 7.5, 1 M NaCl, 1 mM BME, 10 mM Imidazole, and 10% glycerol) with 1 mM PMSF, 5 μg/ml DNase, and one Pierce EDTA-free protease inhibitor cocktail tablet. After lysis by sonication, the clarified lysate was incubated for 30 min with Ni^2+^ resin (0.5 ml per 1 L cell culture). The mixture was added to a gravity column, washed with buffer N2 (20 mM Tris pH 7.5, 1 M NaCl, and 10% glycerol) containing 30 mM imidazole, and eluted with buffer N2 containing 300 mM imidazole. The eluent was incubated with PreScission protease at 4 °C for 2 h to cleave the His tag. The solution was diluted to 167 mM NaCl in MS buffer (20 mM Tris pH 7.5 and 3 mM DTT) and loaded onto a Mono S 10/100 Gl (Cytiva) column equilibrated in MS buffer with 167 mM NaCl. TFAM was eluted with a gradient of MS buffer containing 1 M NaCl, with peak elution near 500 mM NaCl. Peak fractions were concentrated, aliquoted, and stored at −80°C.

TFB2M was first purified by nickel affinity as described for TFAM. The eluent was then incubated with SenP2 at 4 °C for 2 h to cleave the His-SUMO tag. The sample was diluted to 250 mM NaCl in Hep buffer (40 mM Tris pH 7.5 and 3 mM DTT) and loaded onto a 5 ml HiTrap Heparin HP (Cytiva) column equilibrated in Hep buffer containing 250 mM NaCl. TFB2M was eluted with a gradient of Hep buffer containing 1 M NaCl, with peak elution near 500 mM NaCl. Peak fractions were concentrated, aliquoted, and stored at −80°C.

PolRMT WT, 3 KE, and ΔTH were purified by resuspending cells in buffer M1 (1X PBS, 1 M NaCl, 20% glycerol, 5 mM EDTA, 3 mM DTT, and 1 mM PMSF) containing 5 μg/ml DNase and 1 Pierce EDTA-free protease inhibitor cocktail tablet. Cells were lysed by sonication, and the clarified lysate was incubated for 1.5 h with Amylose resin (250 μl per 1 L cell culture). The mixture was loaded onto a gravity column, washed with buffer M2 (40 mM Tris pH 8.0, 1 M NaCl, 20% glycerol, 1 mM EDTA, 5 mM DTT, and 1 mM PMSF), and eluted by incubating overnight with buffer M3 (40 mM Tris pH 8.0, 1 M NaCl, 20% glycerol, 1 mM EDTA, 5 mM DTT, 1 mM PMSF, and 300 mM maltose). The eluent was diluted to 250 mM NaCl in QH buffer (40 mM Tris pH 8.0, 5 mM DTT, 1 mM EDTA, and 10% glycerol) and loaded onto two columns connected in series: 1 ml HiTrap Capto Q column (Cytiva) followed by 5 ml HiTrap Heparin HP column. The columns were equilibrated in QH buffer containing 250 mM NaCl. After loading, the Capto Q column, which contained contaminating nucleic acid, was removed prior to elution. Finally, the heparin column was eluted with a gradient of QH buffer with 1 M NaCl, with peak elution near 400 mM NaCl. Peak fractions were concentrated, aliquoted, and stored at −80 °C.

### *In vitro* transcription initiation assay

DNA templates for *in vitro* transcription assays were generated by annealing complementary oligonucleotides (IDT) (Table S1). Transcription initiation reactions contained 20 nM DNA, 100 nM TFB2M, 60 nM TFAM, and 0 to 300 nM PolRMT variant (presence of TFAM and PolRMT concentrations are indicated in figures) in transcription buffer (40 mM Tris pH 8.0, 10 mM MgCl_2_, 100 μg/ml BSA, 1 mM DTT, and 2 U/μl RNase inhibitor, Murine [NEB]) with 500 μM ATP/CTP/GTP, 10 μM cold UTP, and 33.3 nM ^32^P α-UTP. Reactions were incubated for 30 min at 30°C and stopped by the addition of 2X Urea-PAGE loading buffer (90% formamide, 50 mM EDTA, 0.1% xylene cyanol, and 0.1% bromophenol blue) followed by incubation at 95°C for 10 min. Reactions were resolved by Urea-PAGE on a 20% polyacrylamide gel in 1X TBE buffer with 30 min pre-run followed by a 1.5-h run at 250V. Gels were exposed to phosphor imaging screen overnight, scanned, and quantified using a Sapphire Biomolecular Imager and Azure Spot (Azure Biosystems).

### Promoter-free *in vitro* transcription elongation competence assay

Elongation scaffolds for *in vitro* transcription elongation assays were generated by annealing DNA oligonucleotide pairs with a 5′-FAM-labeled RNA oligonucleotide (IDT) (Table S1). Annealing was performed by mixing the nontemplate strand, template strand, and RNA in a 2:1.5:1 M ratio, heating to 90 °C for 5 min, cooling to 60 °C at a rate of −0.5 °C per min, holding at 60 °C for 20 min, followed by cooling to 12°C at −0.5 °C per min. Transcription elongation reactions contained 20 nM annealed elongation scaffold and 0 to 250 nM PolRMT variant (concentrations are indicated in figures) in transcription buffer with 500 μM ATP/CTP/GTP/UTP. Reactions were incubated for 20 min at 30 °C and stopped by the addition of 2X Urea-PAGE loading buffer followed by incubation at 95 °C for 10 min. Reactions were resolved by Urea-PAGE on a 22.5% polyacrylamide gel in 1X TBE buffer with 30 min pre-run followed by a 1.5-h run at 250V. Gels were scanned and quantified using a Sapphire Biomolecular Imager and Azure Spot.

### Promoter-free *in vitro* transcription elongation single-turnover kinetics assay

Kinetics assays utilized the same elongation scaffold and transcription buffer described in the elongation competence assay. Each reaction contained 100 nM PolRMT, 10 nM elongation scaffold, 100 μM ATP/CTP/GTP/UTP, and a 100-fold excess nonspecific DNA trap. Reactions were incubated at 25°C, and aliquots were quenched at 0, 5, 15, 30, 60, 120, and 300 s. Reactions were resolved, imaged, and quantified as described for the elongation competence assay.

### Double filter binding assay

The 7S RNA substrate for binding assays was *in vitro* transcribed using T7 RNAP, dephosphorylated with calf intestinal phosphatase (NEB), 5′ end-labeled with [γ-^32^P] ATP (Revvity) using T4 polynucleotide kinase (NEB), and purified by denaturing PAGE. 0.1 nM radiolabeled 7S RNA was incubated with PolRMT (0–300 nM) in binding buffer (40 mM Hepes pH 7.5, 100 mM KCl, 20 mM β-ME) at 25°C for 15 min. Reactions were vacuum-filtered through nitrocellulose (0.45 μm, Schleicher & Schuell) and nylon (Whatman) membranes in a dot-blot microfiltration apparatus (Bio-Rad) (52). Membranes were air-dried and quantified by phosphor imaging (Cytiva). The fraction of RNA bound was calculated as the ratio of counts on nitrocellulose to total counts (nitrocellulose + nylon) (53).

### Competition binding assay

Competition binding assays were performed using a slight modification of our double-filter binding assay. Briefly, 50 nM POLRMT and 0.1 nM 5′-end-labeled RNA were incubated in binding buffer at 25 °C for 15 min in the presence of 0.1, 1, 10, or 100 nM unlabeled competitor RNA. Reactions were then vacuum-filtered, and the fraction of labeled RNA bound to POLRMT was determined. Experiments were performed in three independent replicates.

### Cryo-EM sample preparation

The complex was assembled by mixing 3.3 μM TFAM, 5 μM TFB2M, 3.3 μM PolRMT WT, and 3.6 μM HSP -60 to +11 DNA substrate yielding a final concentration for the complex of 1 mg/ml in buffer containing 40 mM Tris (pH 8.0), 10 mM MgCl_2_, 10 mM DTT, and 100 mM NaCl. After 30 min incubation on ice and centrifugation at 17,000 x *g* for 10 min, a 3 μl drop of the complex was spotted onto freshly glow-discharged grids (Quantifoil R 1.2/1.3 Cu 300 mesh grids). Excess sample was blotted using the Vitrobot Mark IV (FEI) with the standard Vitrobot filter paper (Ø55/20 mm [Ted Pella]), with blotting time 2 s and blotting force 3 under 100% humidity at 20 °C. The grids were flash-frozen in liquid ethane and stored in liquid nitrogen.

### Cryo-EM data collection and processing

The dataset of 27,688 movies was collected from Stanford-SLAC Cryo-EM Center and recorded on a Titan Krios G4i electron microscope operated at 300 kV with serial EM (54) (detailed parameters for data collection are summarized in Table 1). Motion correction was performed with MotionCor2 (55), and defocus values were estimated on nondose-weighted micrographs with Gctf (56). For processing of the dataset, reference-free auto-picking (Laplacian-of-Gaussian picking) was performed in RELION-4.0 57, 58. 11,635,618 particles were picked and extracted to pixel size 4.79 Å/pixel and imported to cryoSPARC-4.2 for 2D classification for multiple rounds of 2D classification and subset selection. 7,872,349 particles were used for *ab initio* reconstruction and separated into six classes. Four of those classes were subjected to heterogeneous refinement, where one class showed clear features for PolRMT, TFB2M, and DNA. Particles from this class were further cleaned with 2D classification and split into two classes by *ab initio* reconstruction and heterogeneous refinement. The first class was further processed to yield the HSP without TFAM complex, while the second class was used for the HSP with TFAM complex. Particles from the first class with only density for short upstream DNA were extracted (0.958 Å/pixel) in RELION-4.0 and reimported to cryoSPARC-4.2 for *ab initio* reconstruction into six classes followed by heterogeneous refinement and nonuniform refinement. Particles from the three classes with density for DNA were subjected to heterogeneous refinement with the best map (3.37 Å resolution) and worst map from the previous nonuniform refinement. This yielded 446,280 particles, which were used for CTF refinement and nonuniform refinement resulting in a map with a resolution of 2.86 Å. The output from the nonuniform refinement was then used to generate the final 3DFlex (59) map from 446,000 particles yielding a final resolution of 2.87 Å. Particles from the class with density for longer DNA were extracted (0.958 Å/pixel) in RELION-4.0 and reimported to cryoSPARC-4.2 for *ab initio* reconstruction into six classes followed by heterogeneous refinement and nonuniform refinement. One class yielded a 3.51 Å resolution map with density for the extended DNA, which was used as a template for Topaz (60) training and particle picking. In parallel, this class was subjected to local 3D classification using a focused mask for the extended DNA, which resulted in 2 of 10 classes with density for the DNA in contact with PolRMT. Non-uniform refinement of these two classes produced a 4.13 Å resolution map. Topaz particle picking resulted in 32,940,939 particles which were extracted (0.958 Å/pixel) in RELION-4.0 and reimported to cryoSPARC-4.2 for 2D cleaning resulting in 23,147,478 particles. The particles were then subjected to heterogeneous refinement with the 4.13 Å resolution map combined with five poor maps from previous *ab initio* reconstruction. This produced one class with features of PolRMT, TFB2M, TFAM and extended DNA, which was used for three rounds of iterative *ab initio* reconstruction and heterogeneous refinement. This yielded 200,388 particles, which were used for CTF refinement and nonuniform refinement resulting in a map with a resolution of 3.33 Å. The output from the nonuniform refinement was then used to generate the final 3DFlex map from 200,000 particles yielding a final resolution of 3.35 Å. Phenix auto sharpen (61) was used to aid in model building.

### Model building and refinement

For the HSP with TFAM complex, the previously published structure of the TIC HSP crystal structure (PDB: 6ERQ) was used as an initial model for manually docking into the cryo-EM density map using UCSF Chimera (62). Additionally, an AlphaFold 3 (63) prediction of the mtTIC with the extended HSP substrate was used for modelling the DNA, and the recently published structures of the pre-IC3 (PDB 9GZM) and IC0 (9MN5) on LSP were used for modeling regions of the PolRMT NTE, thumb, and specificity loop and TFB2M NTD. The model was further manually rebuilt in COOT (64) based on electron density and refined in Phenix (65) with real-space refinement and secondary structure and geometry restraints. For the HSP without TFAM complex, the model of the HSP with TFAM complex was used as an initial model for manually docking into the cryo-EM density map using Chimera. An AlphaFold 3 prediction of the mtTIC without TFAM was used to model the linear upstream DNA. As before, the model was manually rebuilt in COOT and refined in Phenix.

### Statistical analysis

All data were graphed and analyzed for significance using GraphPad Prism 10. For transcription initiation reactions with PolRMT titration in Figures 2 and 3, background was subtracted from intensity values and normalization was performed by dividing each intensity by the maximum intensity value from each individual experiment gel.

Significance was determined using a 2-way ANOVA test with Šídák’s multiple comparisons test. For transcription initiation reactions with a single concentration of PolRMT in Figure 4 and Figure S6, background was subtracted from raw values and normalization was performed by dividing each intensity by the intensity value of the sample containing TFAM and WT PolRMT from each individual experiment gel. Significance was determined using a 1-way ANOVA test with Tukey’s multiple comparisons test. For transcription elongation reactions, elongation percentage was calculated by dividing the intensity of the elongation product bands by the total intensity of the lane after subtracting background. Mean and SEM values were determined from 3 independent replicates for all transcription reactions. For filter binding assays, binding measurements were fit to a one-site specific binding model to determine K_D_ values based on 5 independent replicates. To compare the K_D_ values, an extra sum-of-squares F test was performed and the null hypothesis was rejected for *p*-value < 0.05.

### PolRMT, TFAM, and TFB2M sequence conservation analysis

Sequences of eukaryotic orthologues for PolRMT, TFAM, and TFB2M were obtained from OrthoDB and InterPro 43, 66. To generate Tables S4–S6 representing sequence conservation and variance at each residue, the sequences for each set of orthologues were aligned using MAFFT (67). Then, sequences were examined at positions corresponding to the human protein sequence for residue identity and group conservation relative to the human protein, variance, and ratios of conservation and variance between eukaryotes, animals, and mammals. Residue groups were defined as “positive” H/K/R, “negative” D/E, “polar uncharged” C/N/Q/S/T, “hydrophobic” A/G/I/L/M/P/V, “aromatic” F/W/Y, and “gap” if there was no aligned residue.

The PolRMT phylogenetic tree (Fig. S7, *A* and *B*) was constructed by first filtering to remove sequences with greater than 80% sequence identity, then aligned and trimmed using HMM align (68) based on the DNA-dependent RNA polymerase core domain (PF00940) (66). The alignment was further filtered using CD-HIT to remove sequences with greater than 70% identity, yielding a final dataset of 524 sequences. The sequences were further trimmed using trimal (69) and a phylogenetic tree was generated with IQ-TREE (70). The consensus tree was viewed and color-coded with Interactive Tree of Life v7.6 (71). To color-code the tree by features in the N-terminal region, first the sequences on the N-terminal side of the DNA-dependent RNA polymerase core domain (PF00940) were extracted. Sequences containing less than 30 amino acids were color-coded gray for “mtRNAP core only” (mitochondrial RNA polymerase). Then, the extracted sequences were scanned for domain matches with Pfam (72). Sequences that had a match for the canonical RNA polymerase NTD (PF14700), were colored light blue, and those without were colored white. From the sequences with an NTD, sequences containing a PPR domain (PF01535) or a custom PPR domain (query based on human sequence 218–355) were color coded light green and those containing a TPR domain (PF26713) were colored yellow. Finally, the N-terminus of sequences containing a PPR domain were subjected to secondary structure prediction with S4PRED (73), sequences containing a helix of at least 12 residues long, allowing for one nonhelical interruption, and 20% or more K/R residues were annotated as having a “putative tether helix” and colored light red; those without a putative tether helix but containing a stretch of 15 amino acids of 20% or more K/R residues were annotated as having a “basic patch” and colored light purple.

The TFAM sequence logo graphs (Fig. S7*C*) were generated by searching InterPro (66) for sequences containing a high mobility group (HMG) box domain (PF00505) and annotated as a mitochondrial protein. Sequences were then searched for the HMG box domain and the second HMG box (HMG-B) sequence was extracted. The extracted sequences were aligned with HMM align. The aligned sequences were separated into animal and fungal sequences to generate the final sequence logo graphs with Matplotlib (74).

## Data availability

All original data and materials are available upon request. The atomic coordinates for the HSP with TFAM complex and HSP without TFAM complex have been deposited to the Protein Data Bank under accession code PDB: 37FK and PDB: 37FJ, respectively. The corresponding cryo-EM density maps have been deposited to the Electron Microscopy Data Bank under accession code EMDB: 78,147 and EMDB: 78,146, respectively.

## Supporting information

This article contains supporting information.

## Conflict of interest

The authors declare that they have no conflicts of interest with the contents of this article.

## Supporting information

Supporting information 2

**Abbreviations**
HSP, heavy-strand promoter
LSP, light-strand promoter
LSP2, light-strand promoter 2
mtDNA, mitochondrial DNA
MTS, mitochondrial targeting sequence
mtTIC, mitochondrial transcription initiation complex
NP, non-promoter
NTE, N-terminal extension
PolRMT, mitochondrial RNA polymerase
PPR, pentatricopeptide repeat
TFAM, transcription factor A
TFB2M, transcription factor B2
TSS, transcription start site
UBI, upstream backbone interface
