# Supplementary material for: Cryo-EM structures reveal upstream DNA interactions within the mitochondrial transcription initiation complex
Source: J Biol Chem. 2026 Sep 29;302(11):113611. doi: 10.1016/j.jbc.2026.113611 (PMC13734867; doi:10.1016/j.jbc.2026.113611)
Supplement: Supplementary file 1 [file mmc1.docx]

**Cryo-EM Structures Reveal Upstream DNA Interactions within the Mitochondrial Transcription Initiation Complex**

**Authors and affiliations**

Rory E. Sharkey^1^, Caitlin Schroeder^2^, Xiangyu Deng^1^, Jamie Smith^1^, Alfredo J. Hernandez^2^, Yang Gao^1^

1 Department of BioSciences at Rice University, Houston, TX 77005, USA

2 Department of Biology, Tufts University, Medford, MA 02130, USA

Corresponding author: Yang Gao, [yg60@rice.edu](mailto:yg60@rice.edu)

**Running Title**

Structures Reveal mtTIC Upstream DNA Interactions

**Supporting information**

**Figure S1**. Cryo-EM reconstruction of the mtTIC on HSP with and without TFAM, related to **Figures 1** and **4.** (A) Overview of image processing and refinement strategy. Steps performed in RELION are indicated, and all other steps were performed with CryoSPARC-4.2. (B) Representative cryo-EM micrograph. (C) Representative 2D class averages. (D) Gold-standard Fourier shell correlation (FSC) curve of the HSP without TFAM complex between the two half maps, with indicated resolution at FSC=0.143 shown in blue. FSC curve between the refined model and the cryo-EM map, with indicated resolution at FSC=0.5 shown in red. (E) The angular distribution of the particles used in the final 3D reconstruction of the HSP without TFAM complex. (F) Local resolution of the cryo-EM density map of the HSP without TFAM complex. (G) Gold-standard FSC curve of the HSP with TFAM complex between the two half maps, with indicated resolution at FSC=0.143 shown in blue. FSC curve between the refined model and the cryo-EM map, with indicated resolution at FSC=0.5 shown in red. (H) The angular distribution of the particles used in the final 3D reconstruction of the HSP with TFAM complex. (I) Local resolution of the cryo-EM density map of the HSP with TFAM complex.

**Figure S2**. Comparison of our HSP with TFAM structure to other mtTIC structures. (A) Conformation of PolRMT fingers domain from our HSP with TFAM structure in pink compared with the LSP TIC0 structure in gray (PDB: 9MN5) (clenched) and the LSP pre-IC3 with incoming GTP in wheat (PDB: 9R95) (open). From the open to clenched conformation, the fingers domain rotates 20º toward the active site. Structures were aligned by the Cα atoms of the conserved PolRMT palm domain. (B) The TFAM from our HSP complex is rotated by 11º and 15º relative to the LSP TIC0 structure in gray (PDB: 9MN5) and the HSP crystal structure in pale green (PDB: 6ERQ), respectively. Structures were aligned by the Cα atoms of the conserved PolRMT and TFB2M core.

**Figure S3.** Specificity of PolRMT binding to 7S RNA. (A) Quantification of filter binding assays for PolRMT WT to 7S RNA or control RNA. (B) Quantification of competition binding assays with 50 nM WT PolRMT and labeled 7S RNA using unlabeled 7S RNA or control RNA. (C) Quantification of competition binding assays with 50 nM WT PolRMT and 0.1 nM labeled control RNA using unlabeled 7S RNA or control RNA. Mean and SEM were determined from 3 independent replicates.

**Figure S4.** Trajectory of upstream DNA in the HSP with and without TFAM complexes. (A) In the HSP with TFAM complex, TFAM bends the upstream DNA proximal to the active site into a U-turn shape and the more distal upstream DNA extends linearly after the bend. (B) In the HSP without TFAM complex, only a short portion of the upstream DNA is observed, which is linear in the absence of TFAM-induced bending.

**Figure S5.** Electrostatic interactions of the PolRMT tether helix in the HSP with or without TFAM complexes. (A) Electrostatic surface of TFAM and cartoon representation of the PolRMT tether helix in the HSP with TFAM complex. The highly positively charged surface of the tether helix engages the negatively charged helix 3 of the HMG-B box. (B) Electrostatic surface of the positively charged PolRMT tether helix near the negatively charged DNA backbone in the HSP without TFAM complex.

**Figure S6.** The autoinhibitory effect of the PolRMT tether helix follows a similar but less pronounced trend at higher PolRMT concentrations, related to **Figure 4**. Transcription reactions contained 20 nM DNA template, 100 nM TFB2M, 0 or 60 nM TFAM, and 0 or 300 nM PolRMT. (A) Quantification of transcript production from the NP DNA substrate. (B) Quantification of transcript production from the LSP. (C) Quantification of transcript production from the HSP. (D) Quantification of transcript production from the LSP2. Mean and SEM were determined from 3 independent replicates. Significance was determined using a 1-way ANOVA test with Tukey’s multiple comparisons test. Significance: * p<0.05, ** p<0.01, **** p<0.0001.

**Figure S7.** Evolutionary analysis of PolRMT, TFAM, and TFB2M. (A) Phylogenetic tree constructed from an alignment of PolRMT orthologues in eukaryotes based on the highly conserved DNA-dependent RNA polymerase core domain (PF00940). The tree is color-coded based on the region on the N-terminal side of the core domain. Sequences containing only the core are colored light gray. Sequences without a canonical RNA polymerase NTD (PF14700) are colored white, and those with the NTD are colored light blue. Sequences that contain an NTD and a TPR domain are colored yellow, and those with a PPR domain are colored light green. Sequences containing an NTD, PPR domain, and putative tether helix (a helix of at least 12 residues with ≥20% positively charged residues) are colored light red, whereas sequences lacking a tether helix but containing a basic patch (≥20% Lys/Arg residues within a stretch of 15 amino acids) are colored light purple. The indicated domains are shown on the structural model of PolRMT from the HSP with TFAM structure. (B) The same phylogenetic tree as in A, but color-coded by eukaryotic kingdom with plants in blue, fungi in gold, animals in purple, mammals in red, and other eukaryotes in gray. (C) Sequence logo graph of the TFAM high mobility group domain B (HMG-B) helix 3 (residues 206-220) from animals (top) and the aligned residues from the fungal protein ABF2 (bottom). The indicated region of TFAM is shown in orange from the HSP with TFAM structure.

**Table S1**. Oligonucleotides for PolRMT cloning and *in vitro* transcription templates.

| **Oligo**  **Name** | **Description** | **Sequence (5' to 3')** |
| --- | --- | --- |
| HSP 60 NT | HSP NT -60 to +11, mutated from -4 to +3 to create a bubble | CCATCCTACCCAGCACACACACACCGCTGCTAACCCCATACCCCGAACCAACCAAATTATCCCGACACCCC |
| HSP 60 TS | HSP TS -60 to +11 | GGGGTGTCTTTGGGGTTTGGTTGGTTCGGGGTATGGGGTTAGCAGCGGTGTGTGTGTGCTGGGTAGGATGG |
| LSP 40  NT | LSP NT -40 to +20 | GTGTTAGTTGGGGGGTGACTGTTAAAAGTGCATACCGCCAAAAGATAAAATTTGAAATCT |
| LSP 40  TS | LSP TS -40 to +20 | AGATTTCAAATTTTATCTTTTGGCGGTATGCACTTTTAACAGTCACCCCCCAACTAACAC |
| LSP 70 NT | LSP NT -70 to +20 | AGTAGTATGGGAGTGGGAGGGGAAAATAATGTGTTAGTTGGGGGGTGACTGTTAAAAGTGCATACCGCCAAAAGATAAAATTTGAAATCT |
| LSP 70 TS | LSP TS -70 to +20 | AGATTTCAAATTTTATCTTTTGGCGGTATGCACTTTTAACAGTCACCCCCCAACTAACACATTATTTTCCCCTCCCACTCCCATACTACT |
| HSP 40 NT | HSP NT -40 to +20 | ACACCGCTGCTAACCCCATACCCCGAACCAACCAAACCCCAAAGACACCCCCCACAGTTT |
| HSP 40 TS | HSP TS -40 to +20 | AAACTGTGGGGGGTGTCTTTGGGGTTTGGTTGGTTCGGGGTATGGGGTTAGCAGCGGTGT |
| HSP 70 NT | HSP NT -70 to +20 | CAACCCCCGCCCATCCTACCCAGCACACACACACCGCTGCTAACCCCATACCCCGAACCAACCAAACCCCAAAGACACCCCCCACAGTTT |
| HSP 70 TS | HSP TS -70 to +20 | AAACTGTGGGGGGTGTCTTTGGGGTTTGGTTGGTTCGGGGTATGGGGTTAGCAGCGGTGTGTGTGTGCTGGGTAGGATGGGCGGGGGTTG |
| LSP2 40 NT | LSP2 NT -40 to +20 | GAGTCAATACTTGGGTGGTACCCAAATCTGCTTCCCCATGAAAGAACAGAGAATAGTTTA |
| LSP2 40 TS | LSP2 TS -40 to +20 | TAAACTATTCTCTGTTCTTTCATGGGGAAGCAGATTTGGGTACCACCCAAGTATTGACTC |
| LSP2 70 NT | LSP2 NT -70 to +20 | GTACGAAATACATAGCGGTTGTTGATGGGTGAGTCAATACTTGGGTGGTACCCAAATCTGCTTCCCCATGAAAGAACAGAGAATAGTTTA |
| LSP2 70 TS | LSP2 TS -70 to +20 | TAAACTATTCTCTGTTCTTTCATGGGGAAGCAGATTTGGGTACCACCCAAGTATTGACTCACCCATCAACAACCGCTATGTATTTCGTAC |
| NP 70 NT | NS NT -70 to +20  mtDNA sequence 4211-4300 | TATATGATATGTCTCCATACCCATTACAATCTCCAGCATTCCCCCTCAAACCTAAGAAATATGTCTGATAAAAGAGTTACTTTGATAGAG |
| NP 70 TS | NS TS -70 to +20  mtDNA sequence 4211-4300 | CTCTATCAAAGTAACTCTTTTATCAGACATATTTCTTAGGTTTGAGGGGGAATGCTGGAGATTGTAATGGGTATGGAGACATATCATATA |
| ES 42 NT | NT -42 to +10 with -9 to -1 bubble for elongation | GCACTCACAGTCGCATCATAATCGCTCAACGATTAATTATACACGTAGCC |
| ES 42 TS | TS -42 to +10 for elongation | GGCTACGTGTCGCCGGGCGCGTTGAGCGATTATGATGCGACTGTGAGTGC |
| ES 72 NT | NT -72 to +10 with -9 to -1 bubble for elongation | ACTATTCTGCCTAGCAAACTCAAACTACGAACGCACTCACAGTCGCATCATAATCGCTCAACGATTAATTATACACGTAGCC |
| ES 72 TS | TS -72 to +10 for elongation | GGCTACGTGTCGCCGGGCGCGTTGAGCGATTATGATGCGACTGTGAGTGCGTTCGTAGTTTGAGTTTGCTAGGCAGAATAGT |
| ES RNA | FAM-labeled RNA pairs with the TS from -9 to -1 with a 3-nt overhang on the 5' end | /56-FAM/ rUrUrUrCrGrCrCrCrGrGrCrG |
| 7S RNA | 7S RNA for filter binding assays | GGAAAGAUAAAAUUUGAAAUCUGGUUAGGCUGGUGUUAGGGUUCUUUGUUUUUGGGGUUUGGCAGAGAUGUGUUUAAGUGCUGUGGCCAGAAGCGGGGGGAGGGGGGGGUUUGGUGGAAAUUUUUUGUUAUGAUGUCUGUGUGGAAAGCGGCUGUGCAGACAUUCAAUUGUUAUUAUUAUGUCCUA |
| Control RNA | Control RNA for filter binding assays | CCAAAGGACUGCUUACGCUGGCGAAAGGUAAACCAAUCGGUAAGGAAGGUUACUACUGGCUGAAAAUCCACGGUGCAAACUGUGCGGGUGUCGAUAAGGUUCCGUUCCCUGAGCGCAUCAAGUUCAUUGAGGAAAACCACGAGAACAUCAUGGCUUGCGCUAAGUCUCCACUGGAGAACACUUG |
| PolRMT 3KE Fwd | PolRMT Mutation K425E, K428E, K432E | AAGTGGAGCATGCACGCGAGACCCTGAAAACCTTACGTGATCAGT |
| PolRMT 3KE Rev | PolRMT Mutation K425E, K428E, K432E | GTGCATGCTCCACTTCCTCACTCGGCAGGGTCGGTTT |
| PolRMT ΔTH Fwd | PolRMT truncation of residues 122-146 | CCGTTTCAGAGTGGCG |
| PolRMT ΔTH Rev | PolRMT truncation of residues 122-146 | ACGACCACACGGCAC |

**Table S2**. Structure alignment of the HSP with TFAM TIC core to other mtTIC structures. Structures were aligned by the Cα atoms of the conserved PolRMT and TFB2M core.

| PDB ID | Description | Aligned Atoms (#) | RMSD (Å) | Rotation of TFAM (º) |
| --- | --- | --- | --- | --- |
| 6ERQ | HSP TIC0 crystal structure | 1100 | 1.138 | 15.5 |
| 6ERP | LSP TIC0 crystal structure | 1111 | 1.123 | 17 |
| 9MN5 | LSP IC0 | 889 | 0.830 | 11.5 |
| 9MN4 | LSP IC3 with 3-nt RNA | 852 | 0.899 | 14.2 |
| 9GZM | LSP pre-IC3 with 2-nt RNA and incoming GTP | 1157 | 0.832 | 6.8 |
| 9R95 | LSP slipped IC3 with 2-nt RNA, ATP, and incoming GTP | 1129 | 0.697 | 5.9 |
| 9R96 | LSP slipped pre-IC4 with 2-nt RNA, dATP, and incoming GTP | 1139 | 0.813 | 5.7 |
| 9GZN | LSP pre-IC3 with 2-nt RNA, incoming GTP, and no TFAM | 1129 | 0.882 | N/A |
| 9GZO | LSP slipped IC3 with 2-nt RNA, ATP, incoming GTP, and no TFAM | 1137 | 1.031 | N/A |
| 37FJ | HSP without TFAM | 1293 | 0.955 | N/A |

**Table S3**. Structure alignment of the HSP without TFAM TIC core to other mtTIC structures. Structures were aligned by the Cα atoms of the conserved PolRMT and TFB2M core.

| PDB ID | Description | Aligned Atoms (#) | RMSD (Å) |
| --- | --- | --- | --- |
| 6ERQ | HSP TIC0 crystal structure | 1144 | 1.526 |
| 6ERP | LSP TIC0 crystal structure | 1144 | 1.505 |
| 9MN5 | LSP IC0 | 907 | 1.114 |
| 9MN4 | LSP IC3 with 3-nt RNA | 856 | 1.305 |
| 9GZM | LSP pre-IC3 with 2-nt RNA and incoming GTP | 1175 | 1.433 |
| 9R95 | LSP slipped IC3 with 2-nt RNA, ATP, and incoming GTP | 1173 | 1.341 |
| 9R96 | LSP slipped pre-IC4 with 2-nt RNA, dATP, and incoming GTP | 1136 | 1.302 |
| 9GZN | LSP pre-IC3 with 2-nt RNA, incoming GTP, and no TFAM | 1197 | 1.503 |
| 9GZO | LSP slipped IC3 with 2-nt RNA, ATP, incoming GTP, and no TFAM | 1175 | 1.493 |
| 37FK | HSP with TFAM | 1293 | 0.955 |

**Table S4**. Evolutionary conservation of PolRMT residues across eukaryotic orthologues.

**Table S5**. Evolutionary conservation of TFAM residues across eukaryotic orthologues.

**Table S6**. Evolutionary conservation of TFB2M residues across eukaryotic orthologues.
